# Optimized SDS-Based Protocol for High-Quality RNA Extraction from *Musa* spp.

**DOI:** 10.3390/mps8010021

**Published:** 2025-02-19

**Authors:** Kishan Saha, Onyinye C. Ihearahu, L. H. Stevenson Naitchede, Supriyo Ray, George Ude

**Affiliations:** Department of Natural Sciences, Bowie State University, 14000 Jericho Park Road, Bowie, MD 20715, USA; ksaha@bowiestate.edu (K.S.); oihearahu@bowiestate.edu (O.C.I.); lnaitchede@bowiestate.edu (L.H.S.N.); gude@bowiestate.edu (G.U.)

**Keywords:** RNA extraction method, SDS, CTAB, cDNA, *Musa* spp.

## Abstract

The high quantity of polyphenols and polysaccharides present in the tissues of *Musa* spp. often leads to the degradation of nucleic acids, which is why all previously established methods resulted in lesser yield and poor quality of RNA. In this study, we present a modified SDS-based RNA extraction method to improve the quality and yield of RNA from different tissues of *Musa* spp. for downstream applications. The modification of RNA extraction buffer, SDS, heat incubation, and use of LiCl resulted in high-intensity RNA bands and increased RNA yield. The clear ribosomal RNA bands ensured the high quality of intact RNA without genomic DNA contamination, along with A260/A280 and A260/A230 ratios ranging from 1.83 to 2.25, which indicated the high quality of RNA across different banana varieties and tissue types. This method was found to be very effective when RNA was extracted from drought-stressed plants yielding 2.92 to 6.30 µg/100 mg fresh weight with high RNA integrity and quality (RNA IQ) 7.8–9.9 from the different groups of *Musa* tissues. Additionally, the RNA was successfully applied in PCR and quantitative real-time PCR (qRT-PCR), confirming downstream application in gene expression analysis. This method is a reliable and efficient technique for RNA extraction methods like Trizol, NucleoSpin, RNeasy, and CTAB procedures reported so far.

## 1. Introduction

The high-quality DNA-free RNA extraction from different plant species is very critical for diverse applications in molecular biology, including qRT-PCR, reverse transcription-PCR (RT-PCR), cDNA library construction, Northern blotting, in situ hybridization, and gene expression analysis [1,2]. However, the presence of several components such as polysaccharides, secondary metabolites, and polyphenols can significantly hinder the quality and quantity of RNA isolation [3]. The co-precipitation and binding of RNA with these compounds can limit oxidation and degradation, ensuing poor and variable RNA yields [4,5,6]. Subsequently, this can inhibit the downstream reactions that are necessary for preparing sequencing libraries and other molecular biological methods [7,8]. The diverse approaches for extracting high-quality RNA from several plant samples are often required periodically [9]. Many studies have demonstrated that relying on a single protocol for RNA isolation is unlikely to yield adequate results across various plant species [10,11,12]. RNA extraction from several plant species such as *Crocus sativus*, *Gossypium* spp. can be challenging due to being rich in polysaccharides, polyphenolics, quinones, and secondary metabolites; however, modifying the procedure and the extraction buffer resulted in an improved RNA quality and RNA integrity number (RIN) [13,14]. A modified extraction approach has been devised to enhance the yield of RNA from *Picea abies*, addressing challenges related to secondary metabolites and polyphenol concentration [15]. RNA extraction from recalcitrant woody plants such as *Persea americana* (Avocado), *Kandelia candel* (L.) Druce, and *Rhizophora mucronata* Lam., using RNA-based kits, can be complicated; however, optimizing buffer components like CTAB, SDS, and PVP, yields high-quality RNA that is appropriate for sequencing and gene expression analysis [16,17]. Isolation of total RNA using TRIZOL (Invitrogen, Carlsbad, CA, USA) or standard SDS/phenol methods is generally effective for certain plant model organisms like *Arabidopsis*, potato, and *Nicotiana benthamiana* [18]. However, commercial RNA extraction kits often fail to adequately disrupt plant tissues and sequester organic compounds [19]. This limitation is particularly observed when isolating RNA from bananas and plantains (*Musa* spp.), necessitating the development of alternative or modified protocols. Therefore, it is very crucial to adapt or develop efficient RNA extraction methods based on existing techniques to improve yield and quality, especially for *Musa* spp. Bananas and plantains, belonging to the Musaceae family, are cultivated in tropical and subtropical regions such as Asia and Africa and provide an important source of iron, carbohydrates, and various vitamins in developing countries [20,21]. Globally, bananas and plantains are placed as the fourth most important food crops after rice, maize, and wheat [22] and are major staple foods in Asia and Africa [23]. The isolation of high-quality RNA is critical for synthesizing cDNA libraries and conducting sequencing, particularly for *Musa* spp. While several protocols for RNA isolation from different banana tissues have been reported, such as from fruit [24,25], roots [26], and mature leaves [27], there remains a significant gap in efficient RNA extraction methods. Traditional approaches, such as using guanidine isothiocyanate-based buffer, often fall short when extracting RNA from plants rich in polysaccharides and polyphenols, leading to the co-precipitation of sugars with RNA [28,29,30]. Additionally, obtaining high-quality RNA from plant tissues exposed to various environmental stresses can be particularly challenging due to elevated levels of polysaccharides and polyphenols [16,31]. To address these challenges, we have employed different kit-based RNA isolation techniques, including CTAB and SDS methods, specifically adapted for banana plants. Our protocol includes a refined SDS-based RNA extraction protocol developed by [32] and highlights the effectiveness and versatility of the CTAB method [24] for RNA isolation in bananas. This study aims to improve RNA extraction protocols to facilitate molecular research in *Musa* spp. and enhance our understanding of their genetic and physiological responses.

## 2. Experimental Design

**Figure 1:**
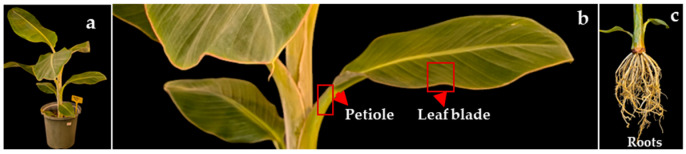
Different developmental stages of *Musa* plants used for RNA isolation. (**a**) The whole *Musa* plant. (**b**) The parts of the petiole and leaf blade are shown in the red box. (**c**) The roots.

Three different genotypes of bananas and plantains were selected for this study. RNA was isolated from the young leaf blades, roots, and petioles of banana plants grown in the Department of Natural Sciences’ greenhouse at Bowie State University (Figure 1). To observe the quality of RNA, the plants were subjected to drought stress for 12 days to compare between control and stress conditions. In a growth chamber set to 26 °C, drought stress was applied to one set of plants [experimental group] by withholding water for 12 days, whereas another set of plants [control group] was regularly watered to maintain the water moisture content of the soil. Our modified SDS method of RNA extraction was compared with other RNA isolation protocols used by the institute and public companies. Additionally, the method was adopted for extracting RNA from the bananas and plantains under stressed and controlled conditions. The following methods of isolation were conducted.

### 2.1. Sample Preparation

The leaf blades, petioles, and roots were collected from three-month-old banana and plantain plants. In the stress vs. control experiment, only the leaf blades and roots were collected and immediately frozen using liquid nitrogen and stored at −80 °C. The tissues were crushed with a mortar/pestle, and 100 mg of tissues were used for each method. Each experiment was replicated 3–4 times to check the reproducibility of the RNA yield used for this study.

### 2.2. Different Methods of RNA Isolation

Before starting, DEPC-treated water was used to prepare all solutions for RNA extraction, and mortars, pestles, glass goods, and plastic wares were autoclaved and then dried.

Trizol method: The total RNA was extracted from different tissue types using TRIzol™ Reagent (Invitrogen, Carlsbad, CA, USA, cat. no.: 15596018) according to the manufacturer protocol and precipitated through isopropanol and dissolved in 0.1% DEPC-treated water.

NucleoSpin RNA Plant kit: The frozen tissues were used for total RNA isolation from the samples as per the manufacturer’s instruction (Macherey-Nagel, Duren, NR-W, Germany, cat. no.: 740949.250). The main basis for this method is using guanidinium thiocyanate, rDNase to remove DNA, and adsorption of RNA to the silica membrane.

RNeasy Plant Mini Kit: The total RNA was isolated based on the protocol described in the manufacturer’s instructions (Qiagen, Hilden, NR-W, Germany, cat. no.: 74904). This kit is based on the combination of guanidine-iso-thiocyanate lysis and silica-membrane purification, ensuring total RNA isolation from the tissues.

CTAB method: The total RNA isolation was conducted as per the protocol by [24]. In this method, the cationic detergent Cetyl-trimethyl ammonium Bromide (CTAB) and LiCl were used to precipitate the RNA.

### 2.3. Qualitative and Quantitative Analyses of RNA Extraction

The quantitative analysis of RNA was conducted by measuring the optical density at 260 nm and 280 nm using the NanoDrop 2000C spectrophotometer (Thermo Fisher Scientific, Wilmington, DE, USA). The RNA quality of the samples was observed by measuring the ratio of A260/A280 nm and A260/A230 nm. Further, the integrity and purity of RNA were evaluated by loading 300 ng/lane using 1% agarose gel electrophoresis (BioRad, Hercules, CA, USA Mini sub gel GT) in TAE buffer. The gel images were captured by the gel documentation system BL (Axygen, cat. no. GDBL1000). The RNA IQ (RNA integrity and quality) value was calculated using the Qubit™ RNA IQ Assay Kits (Invitrogen, USA, cat no. Q33221) with Qubit™ 4 Fluorometer (Invitrogen™, USA, cat no. Q33238). The statistical data analysis was performed using Excel 2016 and GraphPad Prism software V.8.0.1 (244).

### 2.4. Synthesis of cDNA and qRT-PCR

If necessary, the total RNA extracted from different tissue types was treated with DNase I after observing any gDNA contamination in gel documentation for the downstream process. Using SuperScript™ IV Reverse Transcriptase (Invitrogen) and oligo dT primers, 1 µg of RNA was reverse transcribed into cDNA and used for quantitative real-time PCR (qRT-PCR). TB Green Premix Ex Taq II (Takara-RR82WR) was used for qRT-PCR with gene-specific primers to target the desired gene of interest. The following gene primer pairs (MaUBQ2: Macu_Ubq2-Fw: AGAGAGATGCTGCAAAATCCA, Macu_Ubq2-Rv: CCAGCTGTCTGCTCTTGTTCT; MaDREB: Ma8020FP: GGGCTCTTCGACTCTGCTAC, Ma8020RP: TCCCAGCCAGTGGAATTGTC) were used for the qRT-PCR with three technical replicates for each biological sample. Moreover, qRT-PCR was performed with a mixture of 5 µL of SYBR Green master mix, 1 µL of forward primer (10 µM) and reverse primer (10 µM), 10 ng of template cDNA to make the final volume up to 10 µL with nuclease-free water. The qRT-PCR was performed in CFX96 Touch Real-Time PCR Detection System (BioRad-C1000) at 95 °C for 30 s, 40 cycles of 95 °C for 10 s, 56 °C for 15 s, and 72 °C for 20 s. For the melt curve, 65 °C to 95 °C with a 0.5 °C increase per 5 s was used. Relative quantification of the genes was carried out per the method described by [33] and normalized with the UBQ2 gene as a reference [34,35].

### 2.5. Reagents Used in Modified SDS Method

- Tris base, Molecular Biology (Millipore Sigma, Burlington, MA, USA cat. no. 648310)
- Ethylenediaminetetraacetic acid disodium salt, EDTA Na2 (Sigma Aldrich, St. Louis, MO, USA, cat. no. 102075972)
- Polyvinylpyrrolidone, M.W. 50.000, K30 (ACROS cat. no. 227545000)
- Sodium chloride (Fisher Science, Hampton, NH, USA, cat. no. S671-3)
- β-Mercaptoethanol (Fisher Science, cat. no. 03446l)
- Sodium acetate anhydrous (Sigma Aldrich, cat. no. S2889)
- Chloroform (Thermo Scientific, cat. no. J67241.K2)
- DEPC nuclease-free water (Invitrogen, cat. no. AM9920)
- Absolute ethanol MB Grade (Fisher bioreagents, Fair Lawn, NJ, USA cat. no. BP2818)
- Ethidium bromide solution (Sigma Aldrich, cat. no. E1510)
- Acetic acid glacial (Thermo Scientific, cat. no. 036289AE)
- Isoamyl alcohol (Merck Millipore, Co. Wicklow, Ireland, cat. no. 55762055650)
- Phenol (Sigma Aldrich, cat. no. CAS108-95-2)
- Lithium chloride solution (Invitrogen, AM9480)
- RNase Away surface decontamination (MBP, Grand Island, NY, USA, cat. no. 7002)
- Sodium dodecyl sulfate (Sigma Aldrich, cat. no. L3771)
- Agarose MB grade (BioRad, cat. no. 1613101)
- Orange G loading dye (BioRad)
- EDTA 0.5M, pH 8.0 (USB, Cleveland, OH, USA cat. 15694)
- TAE electrophoresis buffer 50X (Thermo Scientific, cat. no. B49)

### 2.6. Stock Solutions

- Tris HCl 1M (pH 8.0): To prepare it, weigh 6.055 g of Tris base and add 30 mL of DEPC-treated water. Adjust pH to 8.0 with conc. HCl and then make the final volume up to 50 mL.
- 30% SDS: Add 15 g of SDS to DEPC water, stir it on a heated stir plate (60 °C) to dissolve the SDS, make up the final volume to 50 mL, and store it at room temperature.
- 0.1 M citrate buffer (pH 4.2): Solution A: Prepare 0.1 M citric acid monohydrate (MW: 210.14 g/mol) (4.202 g in 200 mL), Solution B: Prepare trisodium citrate dihydrate (MW: 294.12 g/mol) (5.88 g in 200 mL), add Solution A (54 mL) + Solution B (46 mL) to make the 0.1 M citrate buffer.
- Sodium acetate (3 M): Weigh 4.92 g of sodium acetate and dissolve it in 12 mL of DEPC water. Adjust the pH to 4.8 with glacial acetic acid. Make the volume up to 20 mL.
- Saturated phenol with citrate buffer (pH 4.2): The pure phenol crystals were melted in a water bath at 65 °C. An equal volume of 0.1 M citrate buffer (pH 4.2) solution was added. At ambient temperature, the solution was mixed thoroughly for 15 min. The phases were allowed to separate at 65 °C and the top layer (remaining non-mixed buffer) was drawn off and discarded. Kept at 4 °C overnight before use.
- Phenol/chloroform/isoamyl alcohol (25:24:1): 50 mL of saturated phenol solution was poured into an amber bottle very carefully. Then, 48 mL of chloroform was mixed in, and 2 mL of isoamyl alcohol was later added. Finally, the solution was mixed thoroughly and kept in the refrigerator for 24 h before use.
- RNA extraction buffer: To prepare 50 mL of RNA extraction buffer, initially add 15 mL of DEPC-treated water to a glass bottle. Then add 5 mL of Tris HCl (100 mM final concentration) from 1 M Tris-HCl (pH 8), 2.5 mL of 0.5 M EDTA-Na_2_ (final concentration 25 mM), 1.25 g of PVP K-30 (final concentration 2.5%), then dissolve the PVP by stirring at 40 °C on a heating plate. After mixing, add 7.305 g of NaCl (final concentration 2.5 M) and make up the volume to 50 mL. Then, 15 µL β-Mercaptoethanol per 750 µL of RNA extraction buffer can be added after autoclaving the RNA extraction buffer.
- SDS (30%): Weigh 30 g of SDS into DEPC-treated water and stir at 60 °C on a heating plate to dissolve the SDS completely, and finally make the volume up to 100 mL.

## 3. Procedure of RNA Isolation

New modified SDS method: RNA isolation was performed using the anionic detergent SDS, low pH phenol-based extraction, and precipitation with LiCl and sodium acetate.

### 3.1. Homogenization of Tissue: Duration: 30 min

1. Immediately transfer the samples into liquid nitrogen from the −80 °C freezer to avoid thawing of the tissue.
2. Take 100 mg of tissue and grind it with a pre-chilled mortar and pestle using liquid nitrogen until a fine powder is observed.
3. Add 750 µL of RNA extraction buffer to 2 mL microcentrifuge tubes (RNase-free) containing 100 mg of finely powdered tissue. Mix the samples vigorously using the vortex until properly mixed.
4. Incubate the homogenate at 60 °C (Thermomixer-Eppendorf) for 10 min after the addition of 0.1 volume of 30% SDS. Briefly vortex the tubes every 5 min. Let the sample cool before proceeding to the next step.
5. Centrifuge (Microfuge 22R Centrifuge, Beckman Coulter) the homogenate at 15,000× *g* for 10 min at room temperature.
6. Carefully transfer the supernatant to a new 1.5 mL microcentrifuge tube (RNase-free) using a micropipette.

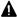

CRITICAL STEP:

1. All plastic materials, mortar, and pestle must be autoclaved. Make sure the working bench and micropipettes are thoroughly cleaned with RNase AWAY. Always wear gloves during the whole RNA isolation procedure.
2. Do not allow the tissues to thaw after freezing in liquid N_2_. Grinding the tissue into a fine powder is very important to obtain high-quality RNA.
3. Do not use more than 100 mg of tissue if you are using 750 µL of RNA extraction buffer.

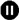

PAUSE STEP: Not applicable for this step.

### 3.2. Extraction of RNA: Duration: 30 min

1. Add an equal volume of ice-chilled phenol (saturated with citrate buffer, pH 4.2), chloroform, and isoamyl alcohol, then mix the sample by inverting the tubes several times.
2. Centrifuge the sample mixture at 15,000× *g* for 5 min at 4 °C.
3. Collect the upper aqueous layer without disturbing the middle whitish layer and transfer it into a new 1.5 mL microcentrifuge tube.
4. Add 250 µL of chloroform and shake vigorously by hand, and centrifuge the mixture at 15,000× *g* for 5 min at 4 °C.
5. Transfer the upper aqueous layer to a new microcentrifuge tube.

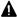

CRITICAL STEP:

1. Add the phenol mixture solution very carefully to avoid direct contact with skin.
2. Do not disturb the lower phase to avoid contamination with proteins, sugars, and organic compounds.

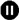

PAUSE STEP: Continue to the next step.

### 3.3. Precipitation of RNA: Duration: 4:30 h

1. Add 3.0 M LiCl solution, mix gently by inversion and incubate at −20 °C for 2 h.
2. Centrifuge the solution at 15,000× *g* for 5 min at 4 °C, a translucent pellet of RNA will be observed. Decant the supernatant very slowly.
3. Add 100 µL of DEPC-treated water with 1/10 volume of 3 M sodium acetate (pH 4.8) and 3 volumes of chilled absolute ethanol. Mix gently and incubate at −80 °C for 2 h.

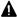

CRITICAL STEP:

1. Proper mixing of the samples governs the enhancement of high RNA yield at each step.
2. The addition of 3 M sodium acetate with ethanol increases the yield, so this is very important step.

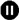

PAUSE STEP

1. Samples can be paused in the last step at −80 °C for better results.

### 3.4. Washing of RNA: Time Duration: 30 min

1. Decant the supernatant after centrifuging the solution at 12,500× *g* for 5 min at 4 °C.
2. Wash the pellet with pre-chilled 80% ethanol solution by centrifuging at 7500× *g* for 5 min at 4 °C.
3. Decant the liquid solution and allow the pellet to air-dry.
4. Dissolve the RNA pellet in DEPC-treated water with an appropriate volume of 20–25 µL and store it at −80 °C immediately for the downstream process.

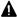

CRITICAL STEP:

1. Over-drying RNA pellets completely may prevent RNA dilution.
2. Care should be taken when decanting the liquid solution.

## 4. Results

Several methods have been reported for isolating RNA from banana tissues; however, the high levels of polyphenols and polysaccharides present in these tissues often complicate the extraction of high-quality RNA. It is crucial to prevent these contaminants from binding to the nucleic acids. Our modified RNA isolation method addresses this challenge, providing a robust approach to obtaining high-quality RNA from banana tissues.

### 4.1. Modified SDS Method Increased Yield and Quality of Total RNA

**Figure 2:**
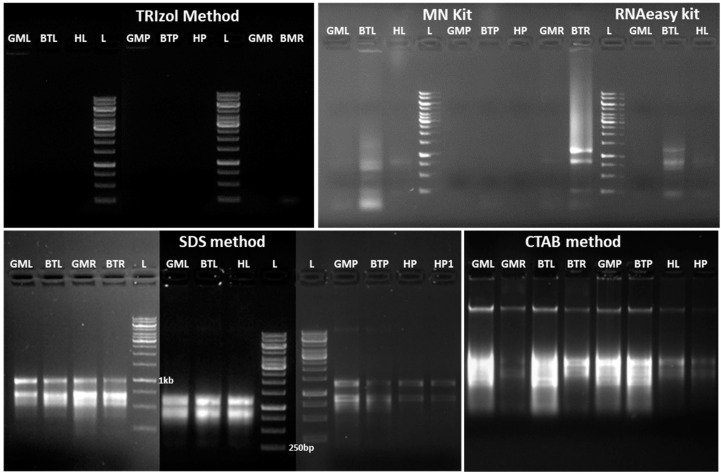
Ethidium bromide-stained 1% agarose gel electrophoresis of isolated RNA from different tissue types extracted using five different methods. The gels were documented using the Axygen gel documentation unit, and further images were cropped using ImageJ software (ImageJ1.54g). Total RNA was isolated at least three times per method. (L: Gene Ruler 1 Kb ladder, GML: Gross Michel leaf blade, BTL: Blue Torres leaf blade, GMR: Gross Michel root, BTR: Blue Torres root. HL: Higa leaf blade, GMP: Gross Michel petiole, BTP: Blue Torres petiole, HP: Higa petiole).

**Figure 3:**
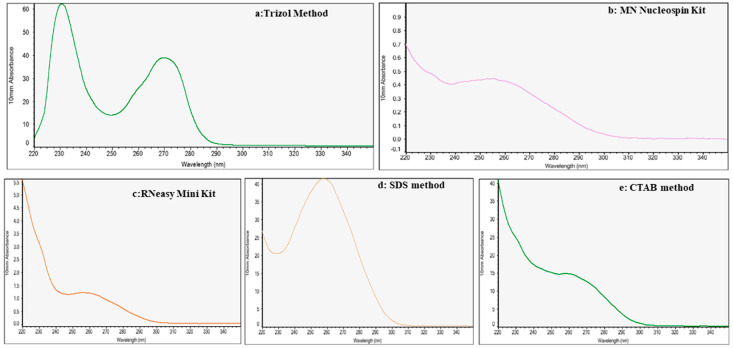
Absorption spectrum of RNA samples isolated from the leaf blades of banana plants using different RNA extraction methods. The five methods, viz. (**a**) Trizol, (**b**) MN NucleoSpin Kit, (**c**) RNeasy Mini kit, (**d**) SDS method, and (**e**) CTAB method, were used for this study. The quality and impurities of the RNA samples can be observed from the spectra, with peaks at 230 (organic contaminants), at 260 (nucleic acids), and at 280 nm (proteins).

The extraction with the modified anionic surfactant SDS is more effective for the isolation of total RNA from banana plants compared to other methods of RNA extraction. The evaluation of total RNA from the different tissue types on agarose gel electrophoresis revealed intact and intense ribosomal RNA, demonstrating that the total RNA extracted by our modified protocol was not degraded (Figure 2). However, the other methods showed either faint or degraded ribosomal RNA smear bands, along with genomic DNA contamination. In the NanoDrop analysis (Figure 3), the ratios of A260/A280 and A260/A230 were close to 2.0 or above (Table 1) for samples extracted from different tissue types of banana varieties.

The highest total RNA yield was 24.54 μg/100 mg FW in Blue torres leaf blades (BTL), whereas the minimum (5.91 μg/100 mg FW) was observed in Higa leaf blades (HL) (Table 2). Thus, the modified SDS method for total RNA extraction from banana is more effective and less time-consuming than the CTAB method.

### 4.2. Effect of RNA Quality When Exposed to Drought Stress

**Figure 4:**
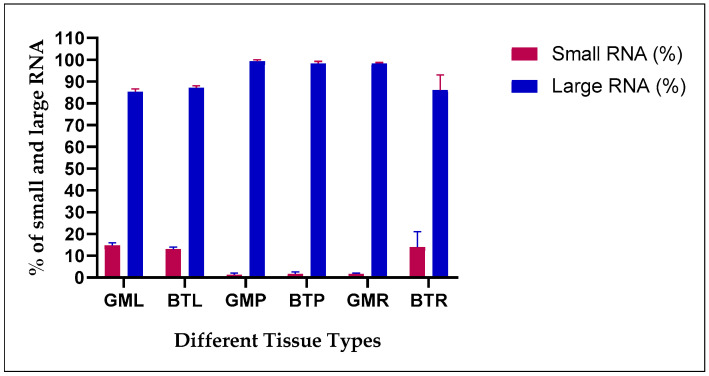
The graphical representation of the percentage of large RNA and small RNA detected using the Qubit fluorometer. (GML: Gross Michel leaf blades, BTL: Blue Torres leaf blades, GMP: Gross Michel petiole, BTP: Blue Torres petiole, GMR: Gross Michel root, BTR: Blue Torres root).

Further, the modified SDS method was tested to check the quality of RNA in leaf blades and roots of two different varieties of banana exposed to drought stress. It was observed that the ratios of A260/A280 and A260/A230 were closer to 2.0, indicating the high purity of RNA. The yield of the total RNA in stressed leaf blades was lower compared to control leaf blades, whereas, in roots, the RNA yield ranged from 2.22 to 3.19 μg/100 mg fresh weight (Table 3). Analysis of the Qubit fluorometer, the RNA integrity and quality (RNA IQ) revealed the high quality of RNA ranging from 7.8 to 9.9 (Table 3), indicating a high percentage of large RNA (Figure 4) isolated by the modified SDS method.

### 4.3. Extracted RNA Using the Modified SDS Method Is Suitable for Downstream Applications

**Figure 5:**
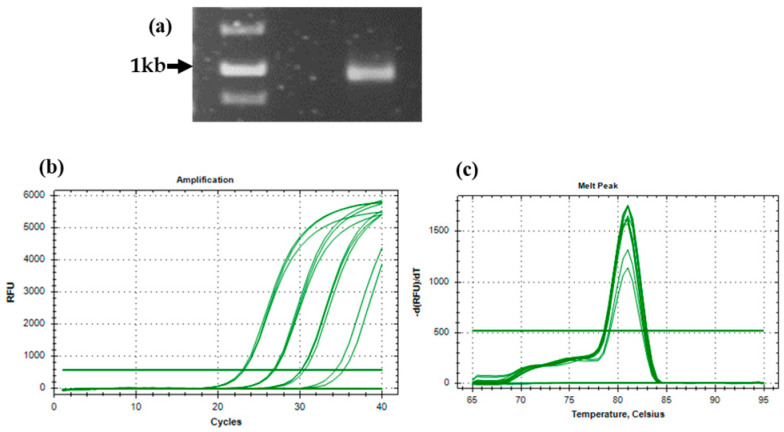
PCR amplification and qPCR efficiency analysis. (**a**) PCR amplicon of full-length *MaDREB* gene from synthesized cDNA. (**b**,**c**) showing the amplification curve and melt peak to determine the qPCR efficiency.

**Figure 6:**
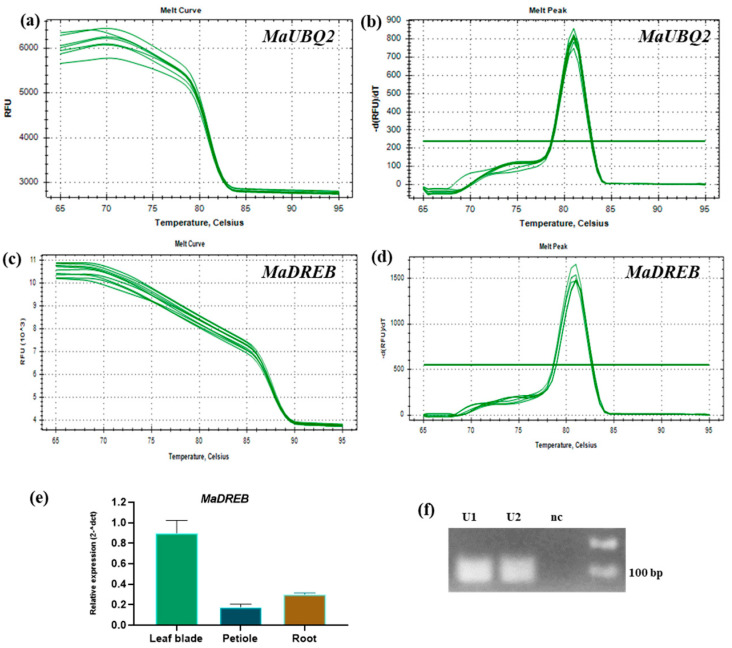
Quantitative real-time PCR assay of RNA extracted from the banana leaf blades using the SDS method. The cDNA was synthesized using RNA extracted from the leaf blades, petiole, and root. (**a**–**d**) showing the qPCR melt curves and peaks of two genes, *MaUBQ2* and *MaDREB.* (**e**) The relative gene expression of *MaDREB* in different tissue types of banana. (**f**) RT-qPCR results of the *MaUBQ2* amplicon (105 bp) shown in 2% agarose gel electrophoresis. U1: Leaf blades and U2: Roots, nc: Negative control.

The PCR amplification of full-length *MaDREB* genes, with 900 bp, from cDNA obtained from RNA using a modified SDS method was successful (Figure 5a). Further, quantitative real-time PCR (qRT-PCR) was performed with the same cDNA. The PCR efficiency was calculated with the *MaUBQ2* gene, and the percentage of efficiency observed was 94.92 (Figure 5b,c), demonstrating the specificity of primers binding to the cDNA synthesized from RNA. In order to check the expression level of one of the MaDREB transcription factors, qPCR was performed along with *MaUBQ2* as the housekeeping gene. Amplification of different samples with a single peak melt curve and lower standard error in technical replicates of MaDREB demonstrated high specificity of primer binding (Figure 6a–f).

### 4.4. Analysis of RNA Quality in Other Methods of Extraction

Absorbance spectra with ratios of A260/A280 and A260/A230 in different methods of RNA isolation showed differences in various tissue types. It was observed that, in the Invitrogen Trizol and MN NucleoSpin kits, the A260/A280 ratios ranged from 1.45 to 2.00 in three varieties of bananas (Table 1). However, the ratios of A260/A230 in these two kits ranged from 0.24 to 1.72, indicating high contaminants such as proteins and polyphenols. Extraction with the RNeasy Plant mini kit failed to isolate RNA from the roots and petioles, although in leaf blades, the A260/A230 was much lower (0.04 to 0.37). Using the CTAB method with different plant tissues revealed a good A260/A280 ratio ranging from 1.81 to 2.10, but the ratio of A260/A230 ranged from 0.21 to 0.35 (Table 1). The absorption spectra analysis of RNA with peak pattern clearly indicates contamination of proteins and polyphenols in other methods of RNA isolation, except for our modified protocol, which indicates a single peak at 260 nm. Evaluation of total RNA yield in different methods revealed significant changes compared to RNA extracted using our modified SDS method. In the Trizol method, RNA yield ranged from 5.82 to 25.32 μg/100 mg FW in different tissue types, but the presence of contamination was observed in the NanoDrop reading as well as in agarose gel electrophoresis (Table 2). Similarly, a lower yield of RNA was reported in NucleoSpin and RNAeasy plant kits, ranging from 0.04 to 1.60 μg/100 mg fresh weight. In addition, using the cationic surfactant, CTAB method, the total RNA yield ranged from 0.96 to 6.79 μg/100 mg FW (Table 2).

## 5. Discussion

Different RNA extraction kits, Trizol (guanidium thiocyanide-based) have been developed for numerous model plant species, such as *Arabidopsis*, tobacco, tomato, rice, and potatoes. However, it can be challenging to isolate high-quality RNA free of contaminants, such as polyphenols and polysaccharides, particularly in *Musa* spp. We sought to establish a more effective method for RNA isolation from various *Musa* spp. tissues using available commercial kits (NucleoSpin, RNeasy), CTAB [24], and SDS methods [32]. Although processes have evolved to improve efficiency and adapt to challenging samples, the core materials have largely remained consistent, with the CTAB protocol being the most widely utilized in several plant species, including *Musa* spp. [24,27,36,37,38]. Our protocol explains an efficient and cost-effective approach for obtaining good-quality RNA for gene expression analysis in *Musa* spp. Recent research supports the failure of commercial kits and only Trizol in isolating high-quality RNA from *Musa* spp. [12]. In some reports, it was observed that even with modifications to the CTAB method, genomic DNA contamination remained a recurring issue, requiring an additional DNase digestion step for removal. Furthermore, certain protocols require the use of large tissue sample quantities (500 mg to 4 g), complicating RNA extraction from plant species, including *Musa* spp. [24,27,36,38,39]. However, in our SDS-based RNA isolation method, consistent DNA contamination was not observed (Figure 2), indicating that DNase digestion can be excluded unless necessary. Previously reported CTAB-based RNA isolation in *Musa* species revealed lower A260/A230 values, indicating phenol contamination [12,24,38]. Interestingly, RNA isolated using our SDS method depicted an A260/A230 ratio ranging from 2.11 to 2.25 (Table 1) and showed minimal contamination from polyphenols and polysaccharides [32,40,41]. RNA isolation procedures from plant tissue should be optimized for simplicity to maximize the yield, purity, and integrity of RNA, ensuring suitability for downstream applications. The quality of RNA obtained from our SDS-based method is suitable for downstream applications, such as cDNA synthesis, RT-PCR, and gene expression analysis, as previously reported in various plant species [32,42,43,44]. Our observation indicated that, in comparison to the control, the leaf blades and the roots of the Gross Michel and Blue Torres plants under drought stress had lower amounts of RNA (Table 3), which showed similar trends as previously reported in different plant species [32,45,46,47]. The exclusion of DNase digestion is notable, as our protocol consistently yielded pure RNA with no genomic DNA contamination (Figure 4) [37]. The primary significance of our protocol lies in the use of high concentrations of SDS (3%) separately and incubation at 60 °C, which effectively disrupts the cell membrane and separates proteins from RNA [32,39,40].

## 6. Conclusions

In conclusion, we have refined a modified SDS-based approach for extracting high-yield and high-quality RNA from various plant tissues of *Musa* spp., devoid of polysaccharides, polyphenols, and other contaminants. Our approach notably improved upon earlier RNA extraction procedures, which yielded low quantities of RNA from *Musa* tissues. The RNA extracted using this SDS-based approach was suitable for downstream applications, as verified through quantitative analysis, gel electrophoresis, and qRT-PCR. Additionally, the established protocol is adaptable for extracting RNA from various *Musa* spp. within a reasonable timeframe.

## Figures and Tables

**Table 1:** Comparative study of A260/A280 and A260/A230 ratios of RNA extracted from different tissue parts of three banana varieties, measured using the NanoDrop™ 2000/2000c Spectrophotometer (Thermo Scientific™).

	GML	BTL	GMR	BTR	HL	GMP	BTP	HP
A260/A280 ratio								
Invitrogen TRIzol	1.61 ± 0.01	1.92 ± 0.02	1.55 ± 0.05	1.84 ± 0.01	1.41 ± 0.01	1.72 ± 0.02	1.67 ± 0.01	1.45 ± 0.01
MN NucleoSpin Kit	2.00 ± 0.01	1.98 ± 0.01	1.82 ± 0.02	1.94 ± 0.02	1.93 ± 0.01	1.55 ± 0.04	1.62 ± 0.02	1.44 ± 0.02
RNeasy Plant mini kit	2.09 ± 0.01	2.10 ± 0.01	na *	na *	2.07 ± 0.01	na *	na *	na *
SDS method	1.96 ± 0.03	2.00 ± 0.08	1.96 ± 0.01	1.93 ± 0.04	1.83 ± 0.03	1.83 ± 0.03	1.94 ± 0.03	1.90 ± 0.02
CTAB method	1.81 ± 0.01	1.99 ± 0.01	1.84 ± 0.01	1.94 ± 0.01	1.84 ± 0.01	2.06 ± 0.03	2.10 ± 0.0	1.90 ± 0.01
A260/A230 ratio								
Invitrogen TRIzol	0.41 ± 0.01	1.72 ± 0.02	0.24 ± 0.02	0.61 ± 0.02	0.33 ± 0.01	0.55 ± 0.01	0.32 ± 0.03	0.36 ± 0.01
MN NucleoSpin Kit	1.05 ± 0.03	1.57 ± 0.02	0.92 ± 0.02	1.33 ± 0.02	0.85 ± 0.03	0.34 ± 0.04	0.54 ± 0.02	0.42 ± 0.02
RNeasy Plant mini kit	0.04 ± 0.01	0.36 ± 0.01	na *	na *	0.37 ± 0.01	na *	na *	na *
SDS method	2.13 ± 0.03	2.20 ± 0.02	2.10 ± 0.08	2.18 ± 0.12	2.20 ± 0.00	2.25 ± 0.03	2.11 ± 0.03	2.12 ± 0.01
CTAB method	0.35 ± 0.01	0.73 ± 0.01	0.39 ± 0.01	0.49 ± 0.03	0.39 ± 0.01	0.35 ± 0.01	0.52 ± 0.00	0.21 ± 0.00

(GML: Gross Michel leaf blades, BTL: Blue Torres leaf blades, GMR: Gross Michel root, BTR: Blue Torres root. HL: Higa leaf blades, GMP: Gross Michel petiole, BTP: Blue Torres petiole, HP: Higa leaves). The values are expressed as the mean ± standard deviation of at least three replicate samples. na * Due to the low quality of the RNA observed, further RNA isolation was not performed for other tissue types.

**Table 2:** Comparative quantification of RNA from different tissue types of banana plants. Five different methods were used to quantify RNA yield (µg/100 mg fresh weight) and measured by the Nanodrop spectrophotometer.

	GML	BTL	GMR	BTR	HL	GMP	BTP	HP
RNA Yield (µg/100 mg fresh weight)								
Invitrogen TRIzol	15.09 ± 0.19	25.32 ± 0.11	8.12 ± 0.05	5.82 ± 0.04	12.70 ± 0.14	36.56 ± 0.15	13.30 ± 0.09	13.58 ± 0.04
MN Nucleo Spin Kit	0.13 ± 0.00	0.48 ± 0.01	0.16 ± 0.02	1.60 ± 0.03	0.20 ± 0.01	0.13 ± 0.01	0.04 ± 0.00	0.35 ± 0.02
Rneasy Plant mini kit	0.11 ± 0.00	0.72 ± 0.01	na *	na *	0.22 ± 0.01	na *	na *	na *
SDS method	14.96 ± 0.12	24.54 ± 0.09	4.05 ± 0.16	4.09 ± 0.05	5.91 ± 0.12	1.268 ± 0.11	0.52 ± 0.05	2.42 ± 0.05
CTAB method	6.19 ± 0.11	6.79 ± 0.09	2.23 ± 0.05	2.80 ± 0.09	2.40 ± 0.04	1.43 ± 0.03	2.67 ± 0.03	0.96 ± 0.02

The values are expressed as the mean ± standard deviation of at least three replicate samples. na * Due to the low quality of the RNA observed, further RNA isolation was not performed for other tissue types

**Table 3:** The total RNA yield, purity, RNA integrity, and quality (RNA IQ) were measured using the Nanodrop spectrophotometer and Qubit 4 Fluorometer (Invitrogen). The RNA was extracted using the SDS method from the control and drought-stressed leaf blades and root samples to compare the RNA yield. Absorption spectra of A260/A280 and A230/A280 were used to measure the quality of the RNA.

SDS Method	RNA Yield (µg/100 mg Fresh Weight)	A260/280 Ratio	A230/280 Ratio	RNA-IQ
GML				
Stress	6.30 ± 0.93	1.96 ± 0.01	1.89 ± 0.06	8.3
Control	11.49 ± 0.76	1.94 ± 0.03	2.03 ± 0.06	8.6
BTL				
Stress	5.58 ± 0.45	1.95 ± 0.03	1.89 ± 0.04	8.8
Control	19.42 ± 0.40	2.07 ± 0.01	2.04 ± 0.02	8.5
GMR				
Stress	2.92 ± 0.19	1.90 ± 0.01	1.90 ± 0.05	9.8
Control	3.19 ± 0.07	1.90 ± 0.02	2.02 ± 0.03	9.9
BTR				
Stress	3.18 ± 0.07	1.90 ± 0.01	2.08 ± 0.08	7.8
Control	2.22 ± 0.03	2.01 ± 0.00	1.88 ± 0.02	8.0

(GML: Gross Michel leaf blades, BTL: Blue Torres leaf blades, GMR: Gross Michel root, BTR: Blue Torres root). The values are expressed as the mean ± standard deviation of at least three replicate samples.

## Data Availability

All data generated and analyzed during this study are presented in this article.

## References

[1] Sasi S., Krishnan S., Kodackattumannil P., Shamisi A.A., Aldarmaki M., Lekshmi G., Kottackal M., Amiri K.M.A. (2023). DNA-Free High-Quality RNA Extraction from 39 Difficult-to-Extract Plant Species (Representing Seasonal Tissues and Tissue Types) of 32 Families, and Its Validation for Downstream Molecular Applications. Plant Methods.

[2] Deepa K., Sheeja T.E., Santhi R., Sasikumar B., Cyriac A., Deepesh P.V., Prasath D. (2014). A Simple and Efficient Protocol for Isolation of High Quality Functional RNA from Different Tissues of Turmeric (*Curcuma longa* L.). Physiol. Mol. Biol. Plants.

[3] Umesh P.P., Ansari M.A., Sridevi G. (2017). An Efficient Method for High Quality RNA Extraction from *Moringa oleifera*. J. Plant Sci..

[4] Gasic K., Hernandez A., Korban S.S. (2004). RNA Extraction from Different Apple Tissues Rich in Polyphenols and Polysaccharides for cDNA Library Construction. Plant Mol. Biol. Rep..

[5] Carpinetti P.D.A., Fioresi V.S., Ignez Da Cruz T., De Almeida F.A.N., Canal D., Ferreira A., Ferreira M.F.D.S. (2021). Efficient Method for Isolation of High-Quality RNA from *Psidium guajava* L. Tissues. PLoS ONE.

[6] Siddique A.B., Rahman S.M.M., Hossain M.A., Hossain M.A., Rashid M.A. (2014). Phytochemical Screening and Comparative Antimicrobial Potential of Different Extracts of *Stevia rebaudiana* Bertoni Leaves. Asian Pac. J. Trop. Dis..

[7] Fang G., Hammar S., Grumet R. (1992). A Quick and Inexpensive Method for Removing Polysaccharides from Plant Genomic DNA. Biotechniques.

[8] Pandey R.N., Adams R.P., Flournoy L.E. (1996). Inhibition of Random Amplified Polymorphic DNAs (RAPDs) by Plant Polysaccharides. Plant Mol. Biol. Rep..

[9] Leh T.Y., Yong C.S.Y., Nulit R., Abdullah J.O. (2019). Efficient and High-Quality RNA Isolation from Metabolite-Rich Tissues of *Stevia rebaudiana*, an Important Commercial Crop. Trop. Life Sci. Res..

[10] Dang T., Bodaghi S., Osman F., Wang J., Rucker T., Tan S.-H., Huang A., Pagliaccia D., Comstock S., Lavagi-Craddock I. (2022). A Comparative Analysis of RNA Isolation Methods Optimized for High-Throughput Detection of Viral Pathogens in California’s Regulatory and Disease Management Program for Citrus Propagative Materials. Front. Agron..

[11] Ahmad J., Baig M.A., Ali A.A., Al-Huqail A., Ibrahim M.M., Qureshi M.I. (2017). Comparative Assessment of Four RNA Extraction Methods and Modification to Obtain High-Quality RNA from *Parthenium hysterophorus* Leaf. 3 Biotech..

[12] Koh R.B.L., Planta J., Encarnacion R.I., Española J.G.G., Aquino V.M., Galvez L.C. (2024). Efficient RNA Extraction Method for Acquiring High-Quality RNA from Various Tissues of the Fiber Crop Abaca, *Musa textilis* Née. Prep. Biochem. Biotechnol..

[13] Wani U.M., Wani Z.A., Koul A.M., Amin A., Shah B.A., Farooq F., Qadri R.A. (2022). Isolation of High-Quality RNA for High Throughput Applications from Secondary Metabolite-Rich *Crocus sativus* L.. BMC Res. Notes.

[14] Mandaliya V.B., Bhatt P., Thaker V.S. (2024). High Quality RNA Extraction Protocol for Polyphenolics-Rich Cotton Tissue. Anal. Biochem..

[15] Singh V.V., Naseer A., Sellamuthu G., Jakuš R. (2024). An Optimized and Cost-Effective RNA Extraction Method for Secondary Metabolite-Enriched Tissues of Norway Spruce (*Picea abies*). Plants.

[16] Nath O., Fletcher S.J., Hayward A., Shaw L.M., Agarwal R., Furtado A., Henry R.J., Mitter N. (2022). A Comprehensive High-Quality DNA and RNA Extraction Protocol for a Range of Cultivars and Tissue Types of the Woody Crop Avocado. Plants.

[17] Nizam A., Kalath H., Kumar A. (2023). A Modified Method for Efficient RNA Isolation from Mangrove Root Tissues Rich in Secondary Metabolites. BioTechniques.

[18] Vicient C.M., Delseny M. (1999). Isolation of Total RNA from *Arabidopsis thaliana* Seeds. Anal. Biochem..

[19] Pelosi J.A., Davenport R., Barbazuk W.B., Sessa E.B., Kuo L. (2024). An Efficient and Effective RNA Extraction Protocol for Ferns. Appl. Plant Sci..

[20] Marriott J., Robinson M., Karikari S.K. (1981). Starch and Sugar Transformation during the Ripening of Plantains and Bananas. J. Sci. Food Agric..

[21] Ghag S.B., Ganapathi T.R., Mérillon J.-M., Ramawat K.G. (2019). Banana and Plantains: Improvement, Nutrition, and Health. Bioactive Molecules in Food.

[22] Moradi Z., Farahani F., Sheidai M., Nejad Satari T. (2017). Somaclonal Variation in Banana (*Musa acuminate colla* Cv. Valery) Regenerated Plantlets from Somatic Embryogenesis: Histological and Cytogenetic Approaches. Caryologia.

[23] Heslop-Harrison J.S., Schwarzacher T. (2007). Domestication, Genomics and the Future for Banana. Ann. Bot..

[24] Asif M.H., Dhawan P., Nath P. (2000). A Simple Procedure for the Isolation of High Quality RNA from Ripening Banana Fruit. Plant Mol. Biol. Rep..

[25] Mbéguié-A-Mbéguié D., Fils-Lycaon B., Chillet M., Hubert O., Galas C., Gomez R.-M. (2008). Extraction and Purification of Total RNA from Banana Tissues (Small Scale). Fruits.

[26] Van Den Berg N., Berger D.K., Hein I., Birch P.R.J., Wingfield M.J., Viljoen A. (2007). Tolerance in Banana to Fusarium Wilt Is Associated with Early Up-regulation of Cell Wall-strengthening Genes in the Roots. Mol. Plant Pathol..

[27] Rodrguez-Garca C.M., Peraza-Echeverra L., Islas-Flores I.R., Canto-Canch B.B., Grijalva-Arango R. (2010). Isolation of Retro-Transcribed RNA from in Vitro Mycosphaerella Fijiensis-Infected Banana Leaves. Genet. Mol. Res..

[28] Liu L., Han R., Yu N., Zhang W., Xing L., Xie D., Peng D. (2018). A Method for Extracting High-Quality Total RNA from Plant Rich in Polysaccharides and Polyphenols Using *Dendrobium huoshanense*. PLoS ONE.

[29] Birtić S., Kranner I. (2006). Isolation of High-quality RNA from Polyphenol-, Polysaccharide- and Lipid-rich Seeds. Phytochem. Anal..

[30] Liao X., Li H., Khan A., Zhao Y., Hou W., Tang X., Akhtar K., Zhou R. (2023). A Simple and Rapid Method for Isolating High-Quality RNA from Kenaf Containing High Polysaccharide and Polyphenol Contents 2020. Biotechniques.

[31] Li B., Wang B., Tang K., Liang Y., Wang J., Wei J. (2006). A Simple and Convenient Approach for Isolating RNA from Highly Viscous Plant Tissue Rich in Polysaccharides. Colloids Surf. B Biointerfaces.

[32] Vennapusa A.R., Somayanda I.M., Doherty C.J., Jagadish S.V.K. (2020). A Universal Method for High-Quality RNA Extraction from Plant Tissues Rich in Starch, Proteins and Fiber. Sci. Rep..

[33] Livak K.J., Schmittgen T.D. (2001). Analysis of Relative Gene Expression Data Using Real-Time Quantitative PCR and the 2−ΔΔCT Method. Methods.

[34] Rego E.C.S., Pinheiro T.D.M., Antonino J.D., Alves G.S.C., Cotta M.G., Fonseca F.C.D.A., Miller R.N.G. (2019). Stable Reference Genes for RT-qPCR Analysis of Gene Expression in the *Musa acuminata*-*Pseudocercospora musae* Interaction. Sci. Rep..

[35] De Castro Costa É., Bastos L.S., Gomes T.G., Miller R.N.G. (2024). Reference Genes for RT-qPCR Analysis in *Musa acuminata* Genotypes Contrasting in Resistance to *Fusarium oxysporum* f. Sp. Cubense Subtropical Race 4. Sci. Rep..

[36] Tong Z., Qu S., Zhang J., Wang F., Tao J., Gao Z., Zhang Z. (2012). A Modified Protocol for RNA Extraction from Different Peach Tissues Suitable for Gene Isolation and Real-Time PCR Analysis. Mol. Biotechnol..

[37] Kiss T., Karácsony Z., Gomba-Tóth A., Szabadi K.L., Spitzmüller Z., Hegyi-Kaló J., Cels T., Otto M., Golen R., Hegyi Á.I. (2024). A Modified CTAB Method for the Extraction of High-Quality RNA from Mono-and Dicotyledonous Plants Rich in Secondary Metabolites. Plant Methods.

[38] Kumara U.M.A., De Costa D.M. (2015). An Efficient Protocol for Isolation of Functional RNA from Peel Tissue of Different Banana (*Musa* spp.) Cultivars for Gene Expression Studies on Anthracnose Development. Trop. Agric. Res..

[39] Liu J.-J., Goh C.-J., Loh C.-S., Liu P., Pua E.-C. (1998). A Method for Isolation of Total RNA from Fruit Tissues of Banana. Plant Mol. Biol. Report..

[40] Huded A.K.C., Jingade P., Mishra M.K. (2018). A Rapid and Efficient SDS-Based RNA Isolation Protocol from Different Tissues of Coffee. 3 Biotech..

[41] George A. (2018). Simple and Efficient Method for Functional RNA Extraction from Tropical Medicinal Plants Rich in Secondary Metabolites. Trop. Plant Res..

[42] Behnam B., Bohorquez-Chaux A., Castaneda-Mendez O.F., Tsuji H., Ishitani M., Becerra Lopez-Lavalle L.A. (2019). An Optimized Isolation Protocol Yields High-quality RNA from Cassava Tissues (*Manihot esculenta* Crantz). FEBS Open Bio.

[43] Impa S.M., Vennapusa A.R., Bheemanahalli R., Sabela D., Boyle D., Walia H., Jagadish S.V.K. (2020). High Night Temperature Induced Changes in Grain Starch Metabolism Alters Starch, Protein, and Lipid Accumulation in Winter Wheat. Plant Cell Environ..

[44] Chang S., Puryear J., Cairney J. (1993). A Simple and Efficient Method for Isolating RNA from Pine Trees. Plant Mol. Biol. Rep..

[45] Sajeevan R.S., Shivanna M.B., Nataraja K.N. (2014). An Efficient Protocol for Total RNA Isolation from Healthy and Stressed Tissues of Mulberry (*Morus* sp.) and Other Species. AJPS.

[46] Camacho-Villasana Y.M., Ochoa-Alejo N., Walling L., Bray E.A. (2002). An Improved Method for Isolating RNA from Dehydrated and Nondehydrated Chili Pepper (*Capsicum annuum* L.) Plant Tissues. Plant Mol. Biol. Rep..

[47] Shahrokhab K., Afshari R.T., Alizade H., Afshari J.T., Javadi G.R. (2008). Compared Two Methods for Isolating RNA from Freezing and Nonfreezing Bread Wheat (*Triticum aestivum* L.) Plant Tissues. Asian J. Plant Sci..

